# Guided Gradual Egg-Tolerance Induction in Hen's Egg Allergic Children Tolerating Baked Egg: A Prospective Randomized Trial

**DOI:** 10.3389/falgy.2022.886094

**Published:** 2022-05-11

**Authors:** Liselot De Vlieger, Lisa Nuyttens, Charlotte Matton, Marianne Diels, Sophie Verelst, Jasmine Leus, Katrien Coppens, Kate Sauer, Ellen Dilissen, Lieve Coorevits, Christophe Matthys, Rik Schrijvers, Marc Raes, Dominique M. A. Bullens

**Affiliations:** ^1^Department of Microbiology, Immunology and Transplantation, Allergy and Immunology Research Group, KU Leuven, Leuven, Belgium; ^2^Department of Pediatrics, University Hospitals Leuven, Leuven, Belgium; ^3^Department of Pediatrics, AZ Maria Middelares, Ghent, Belgium; ^4^Department of Pediatrics, Jessa Hospital, Hasselt, Belgium; ^5^Department of Pediatrics, Imelda Hospital, Bonheiden, Belgium; ^6^Department of Pediatrics, AZ Sint-Jan Hospital, Bruges, Belgium; ^7^Department of Chronic Diseases and Metabolism, Clinical and Experimental Endocrinology, KU Leuven, Leuven, Belgium; ^8^Department of Endocrinology, University Hospitals Leuven, Leuven, Belgium; ^9^Department of General Internal Medicine, University Hospitals Leuven, Leuven, Belgium

**Keywords:** egg allergy, baked egg, heated egg, egg tolerance, egg oral immunotherapy, egg ladder, ovomucoid sIgE, wheat matrix

## Abstract

**Background:**

Over the last few years, studies have shown that the majority of egg allergic children tolerate baked egg (e.g., cake), and that consuming baked egg accelerates the resolution of egg allergy. However, few prospective studies demonstrate the step-wise reintroduction of egg at home after developing baked egg tolerance. Although this could have a positive impact on the children's quality of life and nutrition. Additionally, research supporting the theoretical concept that heating in the presence or absence of wheat causes reduced allergenicity of egg proteins is limited.

**Objective:**

To investigate the clinically most favorable duration of gradual egg-tolerance induction in baked egg tolerant children at home, with regard to complete raw egg tolerance.

**Methods:**

Baked egg tolerant children above 12 months of age were randomly assigned to a short- or long arm protocol. In the short arm, egg-tolerance induction was studied over 18 months compared to 30 months in the long arm. Children were guided through this protocol involving the step-wise introduction of increasingly allergenic forms of egg starting with baked egg offered as cake, followed by hard-boiled egg, omelet/waffle/pancake, soft-boiled egg, and finally raw egg. We hereby designed this protocol based on the influence of thermal processing in the presence or absence of wheat on egg proteins, as investigated by ELISA, SDS-PAGE, and immunoblotting. At inclusion, children either passed an in-hospital cake challenge or had ovomucoid sIgE ≤1.2 kUA/L, which was considered safe for introduction at home.

**Results:**

Gel electrophoresis revealed that the ovalbumin band became weaker with heating, while the ovomucoid band remained stable. In accordance, the IgE-binding to ovalbumin decreased with extensive heating, as opposed to ovomucoid. However, heating in the presence of wheat led to a decreased IgE reactivity to ovomucoid. Of the 78 children in the intention-to-treat group, 39 were randomized to each arm. Fifty-eight children reached the raw egg tolerance endpoint, of which 80% were in the short arm and 69% in the long arm. Within the short arm, the median time to raw egg tolerance was 24 months (95% CI, 21–27 months) compared to 30 months (95% CI, 28–32 months) in the long arm (*p* = 0.005). No grade IV reactions or cases of eosinophilic esophagitis were observed. The short arm was considered to be non-inferior to the long arm.

**Conclusion:**

Our gradual short arm protocol appears to be safe and allows clinicians to guide baked egg tolerant children toward raw egg tolerance at home. The allergenicity of the egg proteins was affected by heating temperature and duration, as well as the presence of wheat.

## Graphical Abstract

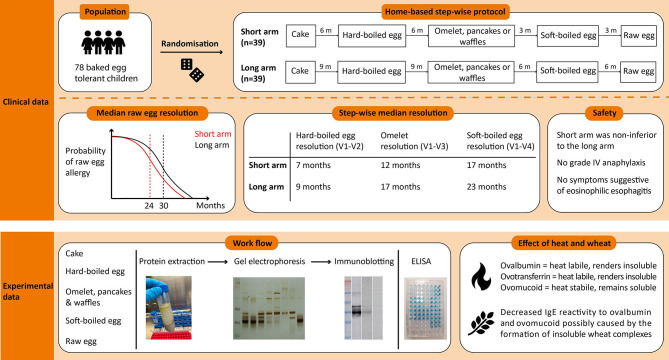

## Introduction

Egg allergy is the second most common food allergy in childhood, affecting 0.5–8.9 % of the pediatric population (1, 2). While the majority of children outgrows their egg allergy by age 16 years, merely 12–50% reaches tolerance between ages 6 to 11 (3, 4). Although strict egg avoidance is part of the standard care, eggs are ubiquitous in most diets making absolute avoidance challenging (5). In turn, this can lead to social isolation and a decreased quality of life (5, 6). However, recent studies have shown that over 70% of egg allergic children can tolerate eggs incorporated in baked foods, such as cookies (7). Leonardet al. (8) showed that when baked egg tolerant children introduced heated egg (e.g., muffin) into their diet, the time needed for tolerance induction was shortened to 50 months as opposed to 78 months in the egg avoidance group. However, prospective studies demonstrating a home-based gradual egg-tolerance induction over time, after baked egg tolerance are limited. To date, only one prospective study evaluating a home-based graduated protocol introducing cookies, followed by pancakes and lightly cooked egg has been reported (9). By introducing each step for at least 3 months, the median age at which allergy to lightly cooked egg resolved decreased to 24 months compared to 78 months in the egg avoidance group (9). Of note, each step was proceeded by a supervised oral food challenge (OFC). In addition, two small retrospective studies showed that by use of an egg ladder, which is a tool used for the step-wise introduction of increasingly allergenic forms of egg at home, children could develop tolerance to scrambled eggs within 8 and 15.5 months (10, 11).

The effect of heat treatment on the allergenicity of the egg plays a key role in achieving baked egg tolerance. Firstly, extensive heating of the egg can reduce the allergenic properties by destroying conformational epitopes (12). Another hypothesis is the “food matrix effect,” which states that the interaction of the egg proteins with carbohydrates, wheat and lipids hampers their bioavailability and digestibility (13). For egg allergy, the egg white proteins ovalbumin and ovomucoid are reported to be the main culprits (13). Ovalbumin is the most abundant protein but is characterized by a low allergenicity due to its heat lability (13). In contrast, the heat-stable ovomucoid is considered the major allergenic protein (13). Accordingly, several studies have evaluated the utility of ovomucoid sIgE levels as a predictor for baked egg tolerance (14–18). At the pediatric allergy department of UZ Leuven, we apply the cut-off of approximately 1.2 kUA/L for ovomucoid sIgE levels, which has shown to be associated with a 97% chance of tolerating baked egg (16).

In this study, we sought to investigate the clinically most favorable duration of a guided gradual induction of egg tolerance in baked egg tolerant children at home. We designed a step-wise egg introducing protocol based on the effect of heating on egg proteins in the presence or absence of the wheat matrix. Second, we studied the relevance of egg sIgE levels to predict tolerance for the next steps.

## Methods

### Egg Protein Extraction

To explore the effect of heating on the allergenicity of the egg proteins, we prepared extracts of increasingly allergenic forms of egg being oven baked egg, hard-boiled egg, omelet, soft-boiled egg and raw egg. Second, we aimed to investigate the role of the wheat matrix in egg preparations equally heated. Accordingly, we prepared extracts of cake, pancakes, and waffles to compare with their supposed corresponding pure egg extract of oven baked egg and omelet, respectively. The details of these egg preparations are provided in Table 1. In brief, the egg preparations were diluted with five volumes of phosphate-buffered saline (PBS) and shaken for 4 h at 4°C. The egg extracts were centrifuged at 4,400 g for 10 min. The pellet was disposed of, and the supernatant was centrifuged at 2,130 g for 10 min. After two extractions, the supernatant was collected and filtered using a 0.2 μm sterile membrane filter. The total protein concentrations were determined by the Bicinchoninic acid assay (Thermofisher).

**Table 1:** Preparation of the egg-containing products.

**Food**	**Ingredients**	**Temperature, heating time, and method**
Oven baked egg	1 egg	Oven: 30' at 150°C (302°F) in a cupcake tin
Cake	4 eggs, 200 g wheat flour*, 200 g plant-based butter, and 200 g sugar	Oven: 35' at 165°C (329°F)
Hard-boiled egg	1 egg	Boiled for 10'
Omelet	2 eggs	Stove top: heated for 3–4' at 120°C (250°F) on both sides
Pancakes	250 g wheat flour*, 500 ml cow's milk**, 3 eggs, and 2 spoons of sugar	Stove top: heated for 2–3' at 180°C (356°F) on both sides
Waffles	250 g wheat flour*, 500 ml cow's milk**, 250 ml sparkling water, 100 g plant-based butter, 2 eggs, and 20 g sugar	Waffle maker: heated for 2–3' at 180°C (356°F)
Soft-boiled egg	1 egg	Boiled for 5'
Raw egg	1 egg	-

* *In wheat allergic children or children with gluten intolerance, wheat flour was substituted by rice flour*.

** *In cow's milk allergic children, cow's milk was substituted by soy milk*.

### ELISA

Commercially available ELISAs (Eurofins) were used to determine the ovalbumin and ovomucoid content of the egg extracts following the manufacturer's instructions. Briefly, protein extracts were diluted 1:20 with the pre-diluted extraction buffer and further diluted depending on the total protein concentration of the extract. One hundred μl of the diluted extracts were incubated for 20 min in the pre-coated 96-well-microplate at room temperature. After washing three times with PBS-0.05% Tween (PBST), 100 μl of the conjugate was added to each well and incubated for 20 min. The wash step was repeated, followed by a 20 min incubation with 100 μl of the substrate solution. The reaction was stopped by adding 0.5 M H_2_SO_4_, and the absorbance was measured at 450 nm. The lower limit of quantification was 25 ppb for ovalbumin and 0.4 ppm for ovomucoid.

### SDS-PAGE and Immunoblotting

Proteins were separated by SDS-PAGE (NuPAGE Bis-Tris 10%, Invitrogen) according to the manufacturer's instructions. The protein samples were preheated for 10 min at 70°C, and 1.5–3 μg of each sample was loaded and separated at 160 V for 1 h. The gel was stained with silver staining (Thermofisher). For immunoblotting, proteins were electrotransferred to a PVDF-membrane (Immobilon-P) at 30 V for 1 h. The membrane was blocked with PBST-5% non-fat dry milk and incubated overnight at 4°C with a 1:8 dilution (PBST-1% BSA) of the patients sera. After washing with PBST, the antigen-IgE complexes were detected by incubating the membrane for 1 h with a 1:1,000 diluted (PBST-1% BSA) mouse anti-human IgE antibody (Genetex, GTX27382) followed by a 2 h incubation with a 1:20,000 diluted goat anti-mouse IgG-HRP antibody (Dako, p0447). Finally, bands were detected by chemiluminescence (Western Lightning Plus ECL-kit, Perkin Elmer) and visualized with the ImageQuant LAS 500 (GE Healthcare Life Sciences).

### Study Design

Ethical approval for this prospective randomized intervention trial (NCT02487420) was provided by the Ethics Committee Research of UZ/KU Leuven. Informed consents were obtained from parents or guardians, with accompanying assents from the child according to the Belgian Legislation. Our primary end point was to define the clinically most favorable time to complete raw egg tolerance, by randomizing children to a 30 and 18 months guided protocol consisting of egg preparations with different proportions of egg allergens in function of the heating (3, 8). Moreover, we wanted to assess whether a period of 18 months of step-wise egg introduction, with regard to complete raw egg tolerance, was not inferior to a total period of 30 months. Secondary end points included defining step-specific risk factors for tolerance failures.

**Figure 1:**
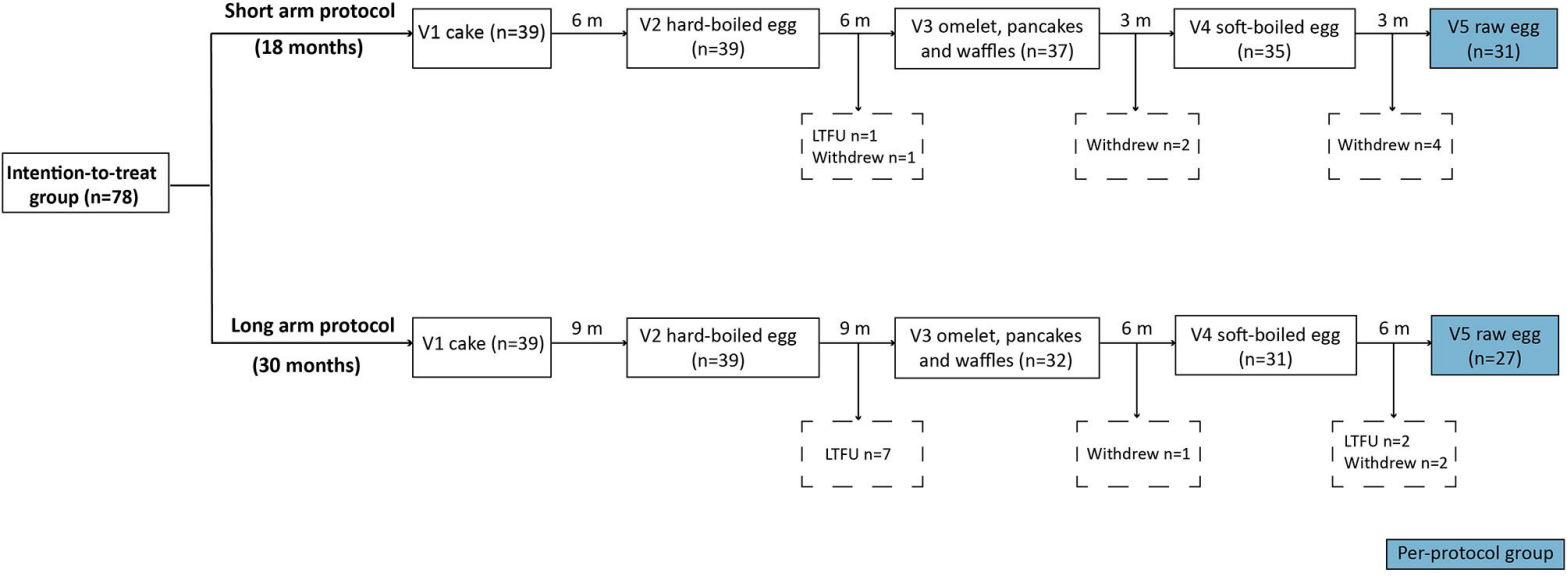
Flow chart of the intention-to-treat group. V, visit; m, month; LTFU, lost to follow-up.

Qualifying subjects were randomized 1:1 by use of a dice (even = 18 months, uneven = 30 months) toward the short- or long arm protocol. In the short arm, the gradual egg-tolerance induction was studied over 18 months compared to 30 months in the long arm. Both protocols involved the step-wise introduction of five egg preparations: baked egg offered as cake at initiation (165°C for 35'), hard-boiled egg (10' boiling) at visit 2, omelet (120°C for 3–4')/ pancakes and waffles (180°C for 2–3') at visit 3, soft-boiled egg (5' boiling) at visit 4, and raw egg (e.g. chocolate mousse, mayonnaise…) at visit 5. By doing so, children were progressively exposed to less heated egg products. Depending on the protocol, the time intervals between each step ranged from 3 to 9 months (Figure 1). In case the child underwent an in-hospital cake OFC, to prove baked egg tolerance at initiation, he or she ingested 6 incremental doses of cake being 1, 2, 4, 8, 16, and 32 g provided with a 15-min dose interval. The recipe of the cake was as follows: 4 eggs, 200 g wheat flour, 200 g sugar, and 200 g plant-based butter baked for 35 min at 165°C. Children who consumed the 63 g cumulative dose of cake (63 g of cake = 1/3 of whole egg = 2 g of egg white protein) without symptoms, were considered to have passed the challenge. The remaining four steps of the protocol were designed for introduction at home without a preceding OFC unless the pediatrician deemed it not safe. After each step, patients were offered a consultation at the pediatric allergy department, as currently done in clinical practice. Provided that there was a clinical indication at the prescheduled visits, peripheral blood was collected and egg sIgE's were measured. When a child experienced symptoms related to the ingestion of an egg preparation in the protocol, the next step was postponed and the current or previous step was repeated for the defined time period. In general, children were advised to consume age-appropriate portions of the egg preparations 2 to 3 times a week to facilitate tolerance toward the next step. A food diary was provided to the parents/caregiver to monitor the frequency of consumption, allergic reactions, medication, or illness.

### Population

Egg allergic children, who tolerated baked egg, were recruited from five pediatric allergy clinics in Flanders (UZ Leuven, AZ Maria Middelares in Ghent, Jessa Hospital in Hasselt, Imelda Hospital in Bonheiden, and AZ Sint-Jan Hospital in Bruges) between July 2015 and December 2018. Inclusion criteria included the following: age between 1 and 18 years, a documented IgE-mediated egg allergy, and a passed cake OFC executed on clinical judgment or ovomucoid sIgE levels below the 1.2 kUA/L or a level that predicts at least a 75% chance to pass the baked egg OFC (14). IgE-mediated egg allergy was defined by a history of a type I hypersensitivity reaction to egg and a positive egg skin prick test (wheal >3 mm) and/or detectable egg sIgE levels (≥0.35 kUA/L). Children were excluded from the study if they were younger than 12 months at the moment of passing the cake OFC, had a grade IV anaphylaxis due to egg ingestion or in case their parents were not able or willing to adhere to the guided egg introductory protocol at home. The CoFAR grading scale for allergic reactions (version 3.0) was used to categorize adverse allergic events (19).

### Intention-to-Treat vs. Per-Protocol Analysis

The intention-to-treat group included the 78 children originally allocated to the short- or long arm protocol after randomization. The per-protocol group included the children (*n* = 58) that completed the step-wise protocol as predefined within due time. Considering that some of the steps in the protocol had to be repeated and/or postponed based on clinical judgment as predefined by the study protocol.

### Statistics

The statistical analysis was performed by GraphPad Prism v.9.2.0 for Windows (La Jolla, CA, USA). The normality of data was evaluated by the D'Agostino and Pearson test. The Fisher exact test and the Mann-Whitney test were used to compare non-parametric data between the two treatment groups. Probability of step-wise egg-tolerance was estimated with the Cox proportional hazard regression model. We used receiver operating characteristic (ROC) curve analysis to determine cut-off levels, sensitivity, and specificity for egg sIgE's in function of developing raw egg tolerance. The non-inferiority margin was set at a 10% difference in failure rate for the development of raw egg tolerance. A *p*-value below 0.05 was considered statistically significant.

## Results

### Egg Protein Content of Egg-Containing Preparations

The ovalbumin and ovomucoid content of the pure egg extracts (oven baked egg, hard-boiled egg, omelet, soft-boiled egg, and raw egg) and the processed extracts (cake, waffles, and pancakes) were analyzed by ELISA and depicted in Table 2. In the pure egg extracts, both ovalbumin and ovomucoid content gradually decreased with thermal processing, with the exception of the ovalbumin content in hard-boiled egg. In accordance, the ovalbumin/total protein (ovalbumin + ovomucoid) ratio increased with lower heating temperature and duration, while the ovomucoid/total protein ratio decreased. Hereby, the ovalbumin/total protein ratio of cake exceeded that of oven baked egg, whereas waffles and pancakes had a higher ovomucoid/total protein ratio as compared to omelet. The egg protein content of the processed egg extracts were significantly lower compared to their supposed corresponding pure egg extract.

**Table 2:** Ovalbumin and ovomucoid content of the egg extracts by ELISA.

**Egg extract**	**OVA**	**OVM**	**OVA/OVA+**	**OVM/OVA+**
	**(μg/mL)**	**(μg/mL)**	**OVM (%)**	**OVM (%)**
Oven baked egg	464.75	5500	7.79	92.21
Cake	19.675	10	66.30	33.70
Hard-boiled egg	13.4	9780	0.14	99.86
Omelet	5776	12400	31.78	68.22
Waffles	1.334	850	0.16	99.84
Pancakes	20.42	770	2.58	97.42
Soft-boiled egg	19840	34000	36.85	63.15
Raw egg	40950	48000	46.04	53.96

*OVA, ovalbumin; OVM, ovomucoid*.

### Effect of Heating on the Protein Pattern of Egg Proteins

**Figure 2:**
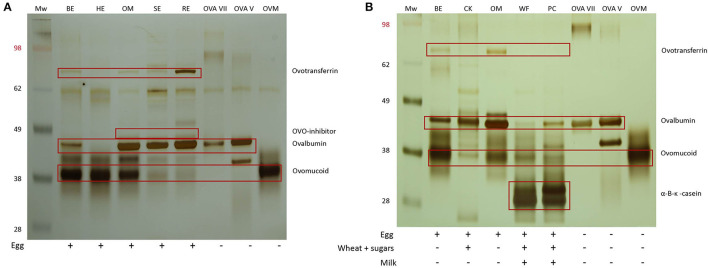
**(A)** SDS-PAGE analysis of the five pure egg extracts followed by silver staining. MW, SeeBlue™ Pre-stained Protein Standard (Thermofisher, LC5625), BE, oven baked egg; HE, hard-boiled egg; OM, omelet; SE, soft-boiled egg; RE, raw egg; ovalbumin (OVA) grade VII (Sigma), OVA grade V (Sigma), ovomucoid (OVM) type III free of ovo-inhibitor (Sigma). **(B)** SDS-PAGE pattern of the pure egg extracts of oven baked egg and omelet and their corresponding processed extracts of cake and waffles/pancakes, respectively. CK, cake; WF, waffles; PC, pancakes.

Ovomucoid was observed in all the egg extracts, indicating that this protein remains soluble and detectable after heat treatment, consistent with its known heat resistant nature (Figure 2A) (20). Although the molecular weight of ovomucoid is set at 28 kDA, the protein appeared at 30–40 kDA due to the presence of carbohydrate chains. The lower intensity of the ovomucoid band in soft-boiled egg and raw egg could be attributed to the overall higher protein content, and therefore lower ovomucoid/total protein ratio. Ovotransferrin (76.6 kDA) was only visible in the raw egg extract, indicating that heating either results in the destruction of ovotransferrin and/or abolishes its water solubility, consistent with its known heat labile nature (20). The same applied for the heat labile ovalbumin fraction (45 kDA) which was stronger in the less/non-heated extracts of omelet, soft-boiled egg, and raw egg, while increasing heating time and temperature led to a weaker or non-detectable band in oven baked egg and hard-boiled egg (Figure 2A). The weak protein fraction above the ovalbumin band of omelet, soft-boiled and raw egg is assumed to be ovo-inhibitor (46.5 kDA).

To investigate the influence of the food matrix, pure egg extracts of oven baked egg and omelet were compared with their corresponding processed extracts of cake and waffles/pancakes, respectively (Figure 2B). The ovalbumin and ovomucoid fractions were visible in all the extracts, but to a lesser extent in some of the processed extracts of cake, waffles, and pancakes. A weak band corresponding to ovotransferrin was visible in oven baked egg and omelet but was not detectable in cake, waffles, and pancakes. This could indicate the formation of wheat and egg protein complexes, which could have influenced the protein pattern and/or separation.

### Effect of Heating on IgE Binding to Egg Proteins

**Figure 3:**
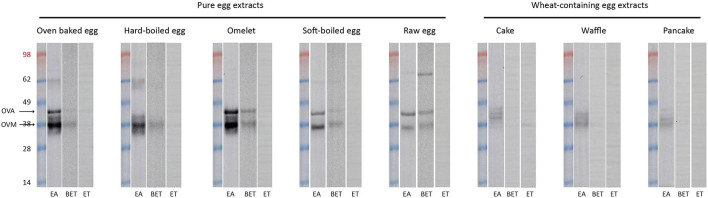
Immunoblots of the pure and processed egg extracts with sera of an egg allergic (EA) child, a baked egg tolerant (BET) child, and at present complete egg tolerant (ET) child who had a grade III reaction toward egg at initial presentation. At the time of the experiment the egg sIgE levels were as followed, *for the EA (age: 13 m, girl)*: IgE egg white 16.30 kUA/L– IgE ovalbumin 12.30 kUA/L– IgE ovomucoid 8.42 kUA/L, *for the BET (age: 11 year, boy)*: IgE egg white 1.27 kUA/L– IgE ovalbumin 1.36 kUA/L– IgE ovomucoid 0.44 kUA/L and *for the ET (age: 3 year, girl):* IgE egg white 0.20 kUA/L– IgE ovalbumin 0.20 kUA/L– IgE ovomucoid 0.10 kUA/L. Of note, silver staining was not performed in parallel but presence of the respective proteins was inferred from earlier SDS-PAGE analysis of the egg extracts as indicated in Figure 2.

We performed immunoblotting with sera from an egg allergic child (EA), a baked egg tolerant (BET) child, and at present complete egg tolerant (ET) child (Figure 3). We used a spectrum of heat-treated pure egg extracts (oven baked, hard-boiled egg, omelet, soft-boiled, and raw egg) and wheat-containing egg extracts (cake, waffles, pancakes). The immunoreactivity was mainly found against ovomucoid in the pure egg extracts, with a higher intensity in the EA child compared to the BET child. As for ovalbumin in the pure egg extracts, IgE binding was influenced by thermal processing and only detected in omelet, soft-boiled egg, or raw egg for the BET child, while remaining detectable for all pure egg extracts for the EA child. IgE binding to ovalbumin was not detected for the hard-boiled egg extract in either participant, possibly because of its absence in this assay (Figure 2A, lane HE). Considering the wheat-containing preparations of cake, waffles/pancakes, there was a significantly weaker to absent IgE binding to ovomucoid or ovalbumin for both the EA and BET child, compared to their respective pure extracts, being oven baked egg and omelet, respectively. However, this could be explained in part by the lower amount of ovomucoid or ovalbumin that was retrieved in these wheat-containing preparations (Figure 2B), except for ovalbumin in cake. Finally, no immunodetection was observed in the complete ET child.

### Study Population

Eighty-seven egg allergic children fulfilled the inclusion criteria. Of them, five were lost to follow-up (LTFU) after visit 1, one child refused to eat cake and three children had gastro-intestinal complaints during the cake OFC and were considered to be not fully baked egg tolerant. Ultimately, 78 children were included in the intention-to-treat group of whom 39 were randomized to the 18 months protocol (short arm) and 39 to the 30 months protocol (long arm) (Figure 1). In the short arm, one child was LTFU after step two, while seven children dropped out of the study after step two (*n* = 1), three (*n* = 2) and four (*n* = 4), respectively. In the long arm, nine children were LTFU after step two (*n* = 7) and four (*n* = 2), whereas three children withdrew from the study after step three (*n* = 1) and four (*n* = 2). The most common dropout reasons were the child being a difficult eater, parents considering the intake of raw egg unnecessary, and/or a general increase in eczema. Only one child from the short arm stopped the study early due to a grade III reaction to raw egg at home. Ultimately, 58 children completed the five-step protocol including 31 of the short arm and 27 of the long arm.

### Baseline Characteristics

The demographic and clinical characteristics of the intention-to-treat group are described in Table 3. The median age at enrollment was 3 years (IQR 1.75–4), with over half of the children being boys. Based on medical records, most children were diagnosed with egg allergy at the age of 10 months (IQR 7–15) and had a grade I reaction toward egg at initial presentation. The median time between their initial reaction to egg and enrollment in the study was 1.5 years (IQR 0.53–3), with a significantly shorter time period of 1.2 years (IQR 0.4–2.45) in the long arm compared to 2 years (IQR 1–3.5) in the short arm. Fifty-one children suffered from multiple food allergies, with a concomitant allergy to cow's milk, peanuts, and/or treenuts being the most common. Concerning atopic diseases, 77% of the children were affected by atopic dermatitis while only a minority suffered from allergic asthma (35%) and/or allergic rhinitis (27%). At the study entry, the median egg white sIgE levels were 1.02 kUA/L (IQR 0.45–2.40), with egg component sIgE levels for ovomucoid of 0.42 kUA/L (IQR 0.10–1.51) and for ovalbumin of 0.32 kUA/L (IQR 0.10–1.53). The two treatment groups were overall similar with respect to demographics, atopic diseases, egg sIgE levels and other clinical characteristics with the exception of the interval between the index reaction and enrollment.

**Table 3:** Baseline demographic and clinical characteristics of the intention-to-treat group.

	**Total** **(*n* = 78)**	**Short arm** **(*n* = 39)**	**Long arm** **(*n* = 39)**	***p*** -value*
**Gender**				0.48
Boy	50	27	23	
Girl	28	12	16	
**Age at enrolment (years)**	3 (1.75–4)	3 (2–5)	2 (1–3)	0.07
**Age diagnosis egg allergy (months)**	10 (7–15)	9 (7–12)	11 (6.50–19)	0.62
**Time between initial reaction to egg and study enrolment (years)**	1.5 (0.53–3)	2 (1–3.5)	1.2 (0.4–2.45)	0.04
**Grading initial egg allergic reaction**				0.58
Grade I	56	26	30	
Grade II	3	2	1	
Grade III	19	11	8	
**Multiple food allergies**				0.64
Yes	51	27	24	
No	27	12	15	
**Allergic asthma**				0.64
Yes	27	12	15	
No	51	27	24	
**Allergic rhinitis**				0.61
Yes	21	9	12	
No	57	30	27	
**Atopic dermatitis**				0.06
Yes	60	26	34	
No	18	13	5	
**House dust mite allergy**				0.82
Yes	30	14	16	
No	48	25	23	
**Tree pollen allergy**				0.45
Yes	22	9	13	
No	56	30	26	
**Grass pollen allergy**				0.33
Yes	25	10	15	
No	53	29	24	
**Cat allergy**				0.10
Yes	28	10	18	
No	50	29	21	
**Total IgE (kU/L)**				
Median (IQR)	185.2	114	225	0.75
Min, max	(38.23–675.2) 4–4893	(40.20–741) 4–3438	(32.10–653.2) 4–4893	
**sIgE egg white (kUA/L)**				
Median (IQR)	1.02	1.01	1.02	0.76
Min, max	(0.45–2.40) 0.10–14	(0.45–2.00) 0.10–14	(0.32–2.74) 0.10–10	
**sIgE egg yolk (kUA/L)**				
Median (IQR)	0.15	0.15	0.15	0.58
Min, max	(0.10–0.46) 0.10–8.55	(0.10–0.57) 0.10–8.55	(0.10–0.38) 0.10–2.23	
**sIgE ovomucoid (kUA/L)**				
Median (IQR)	0.42	0.68	0.38	0.69
Min, max	(0.10–1.51) 0.10–8.55	(0.10–1.76) 0.10–7.82	(0.10–1.18) 0.10–8.55	
**sIgE ovalbumin (kUA/L)**				
Median (IQR)	0.32	0.18	0.52	0.52
Min, max	(0.10–1.53) 0.10–9.60	(0.10–1.27) 0.10–9.60	(0.12–1.83) 0.10–5.39	

*Data are presented as absolute values expressed as median (IQR)*.

* *The Mann-Whitney or Fisher-exact test were utilized where appropriate*.

### Step-Specific Failures and Protocol Deviations

A total of 15 children in the intention-to-treat group had to postpone a next step in the protocol and repeated the current or previous step for the defined time period as stated by the protocol (Table 4). Of these, 11 experienced an allergic hypersensitivity reaction during step one (*n* = 2), two (*n* = 6), four (n = 1), or five (*n* = 2), while four children delayed a step due to a general increase in eczema, parents' choice, and/or low frequency intake. The allergic reactions consisted of facial edema, urticaria, itch, cough, abdominal cramps, diarrhea, and/or vomiting. However, nine of the eleven children were ultimately able to proceed to the next steps of the protocol without symptoms, while two children were LTFU. The baseline median egg white sIgE levels of these 11 children who experienced an allergic reaction were 1.9 kUA/L (IQR 0.45–2.18) with an ovomucoid sIgE level of 0.75 kUA/L (IQR 0.10–2.95) and an ovalbumin sIgE level of 0.12 kUA/L (IQR 0.08–1.89), which did not significantly differ from the total group. The majority of these 11 children experienced a grade I (*n* = 7) or grade III (*n* = 3) reaction toward egg at initial presentation.

**Table 4:** Step-specific failures in the short- and long arm.

**Step**	**Short** **arm**	**Failure**	**Long** **arm**	**Failure**
Step 1: baked egg offered as cake	1	Grade III reaction cupcake	1	Grade I reaction cake
Step 2: hard-boiled egg	5	Increase in eczema (*n* = 1) Grade III reaction HE (*n* = 3) Grade II reaction HE (*n* = 1)	2	Grade III reaction HE (*n* = 1) Grade II reaction HE (*n* = 1)
Step 3: omelet, pancakes and waffles	0		0	
Step 4: soft-boiled egg	4	Choice parents (*n* = 2)* Grade I reaction SE (*n* = 1) Low frequency intake SE (*n* = 1)	0	
Step 5: raw egg	1	Grade III reaction ice cream (*n* = 1)	1	Grade II reaction chocolate mousse (*n* = 1)

*HE, hard-boiled egg; SE, soft-boiled egg*.

* *The parents decided themselves, without medical indication, to repeat this step in the protocol and postpone the introduction of raw egg*.

Concerning accidental ingestions, 23 children in the short arm and 23 children in the long arm reported accidental exposure to less heated egg in processed foods. Of these children, only a minority (16/46) experienced grade I (12/16) or III (4/16) reactions and ultimately all of them continued with the gradual egg introduction. In addition, 22 children speeded up the process of tolerance development by accidentally or intentionally skipping steps or as a result of harmless accidental exposure, of which only one child experienced a grade I reaction. None of the children developed symptoms suggestive of eosinophilic esophagitis (EoE) during the study.

### Guided Step-Wise Egg-Tolerance Induction

**Figure 4:**
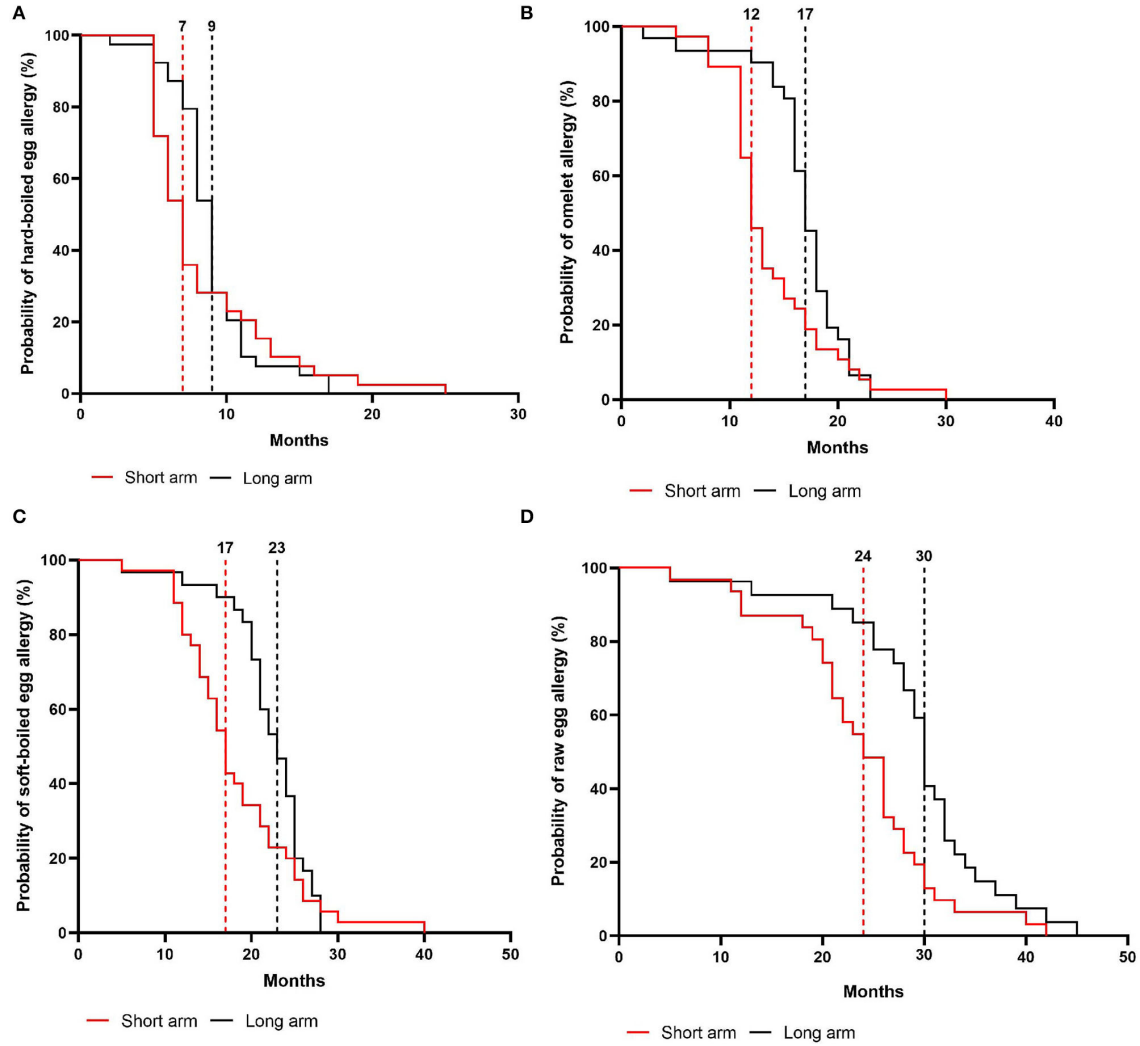
Cox regression analysis of the step-wise egg-tolerance induction over time in the intention-to-treat group. **(A)** hard-boiled egg resolution over time (SA: *n* = 39, LA: *n* = 39) **(B)** omelet resolution over time (SA: *n* = 37, LA: *n* = 32) **(C)** soft-boiled egg resolution over time (SA: *n* = 35, LA: *n* = 31) **(D)** raw egg resolution over time (SA: *n* = 31, LA: *n* = 27). SA, short arm protocol; LA, long arm protocol.

**Figure 5:**
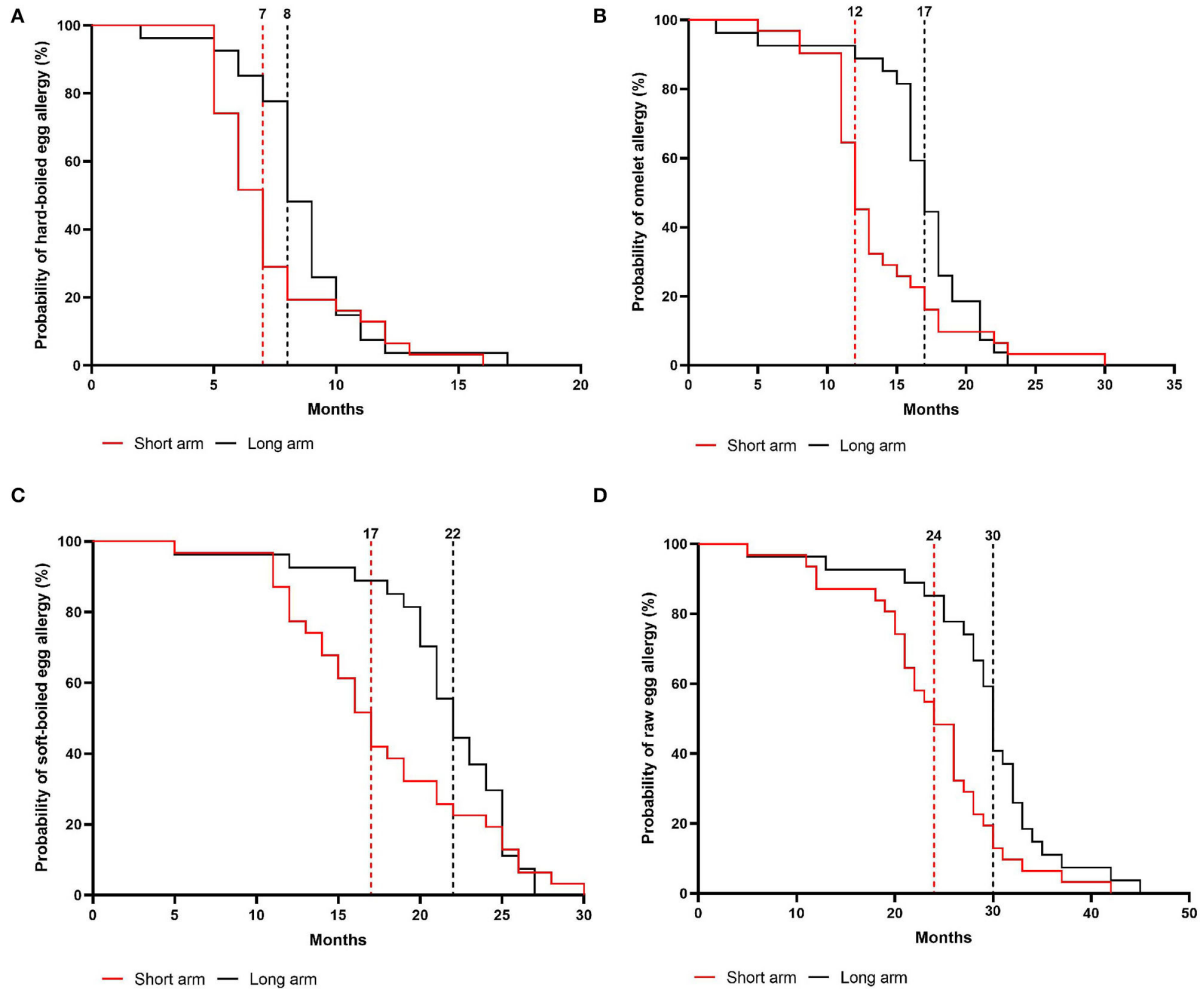
Cox regression analysis of the step-wise egg-tolerance induction over time in the per-protocol group. **(A)** hard-boiled egg resolution over time (SA: *n* = 31, LA: *n* = 27, *P* = 0.08) **(B)** omelet resolution over time (SA: *n* = 31, LA: *n* = 27, *P* = 0.02) **(C)** soft-boiled egg resolution over time (SA: *n* = 31, LA: *n* = 27, *P* = 0.12) **(D)** raw egg resolution over time (SA: *n* = 31, LA: *n* = 27, *P* = 0.005). SA, short arm protocol; LA, long arm protocol.

**Figure 6:**
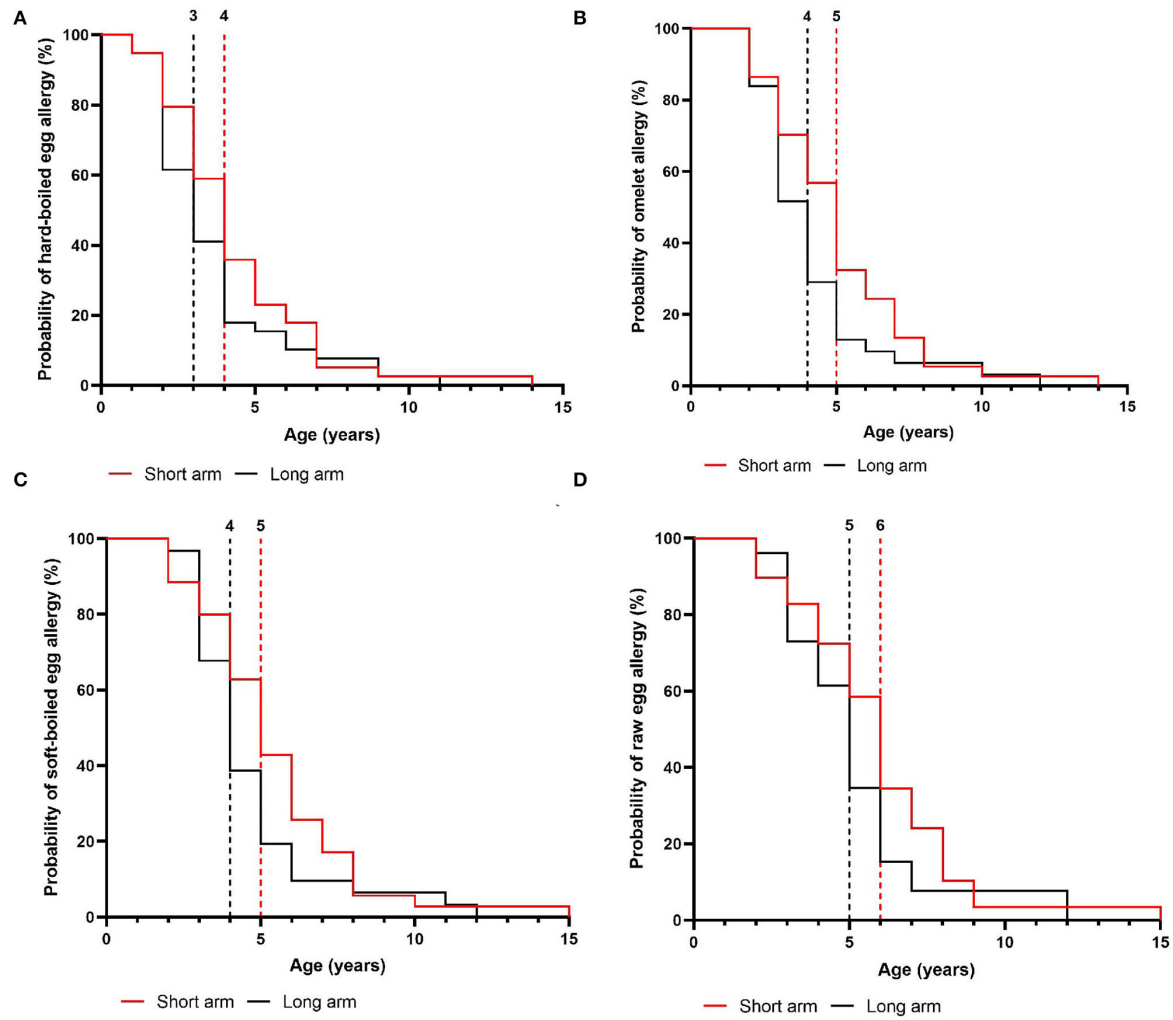
Age of the children at the time of acquiring step-specific tolerance in the intention-to-treat group. **(A)** hard-boiled egg resolution over time (SA: *n* = 39, LA: *n* = 39) **(B)** omelet resolution over time (SA: *n* = 37, LA: *n* = 32) **(C)** soft-boiled egg resolution over time (SA: *n* = 35, LA: *n* = 31) **(D)** raw egg resolution over time (SA: *n* = 31, LA: *n* = 27). SA, short arm protocol; LA, long arm protocol.

In the intention-to-treat group, children from the short arm developed hard-boiled egg tolerance over a median time-period of 7 months (95% CI, 6–8 months) compared to 9 months (95% CI, 8–9 months) in the long arm (*p* = 0.36, Figure 4A). Subsequently, the total time needed to develop omelet tolerance was significantly faster in the short arm (12 months, 95% CI 11–14 months) than in the long arm (17 months, 95% CI 16–18 months; *p* = 0.01, Figure 4B). The soft-boiled egg resolution took only 17 months (95% CI, 15–21 months) in the short arm while 23 months (95% CI 21-25) in the long arm (*p* = 0.06, Figure 4C) without reaching significance. Finally, the median time to complete raw egg tolerance was 24 months (95% CI, 21–27 months) in the short arm compared to 30 months (95%CI, 28–32 months) in the long arm (*p* = 0.005, Figure 4D). Similar results were obtained in the per-protocol group (Figure 5). At the time of acquiring complete egg tolerance, children in the short arm were on average 6 years (IQR 4–7 years) of age and those in the long arm 5 years (IQR 3–6 years) of age (Figure 6).

Overall, 80% of the children in the short arm vs. 69% in the long arm developed raw egg tolerance. Based on the non-inferiority margin, which was set at a 10% difference in failure rate for the development of raw egg tolerance, we can conclude that the short arm (20% failure) is non-inferior to the long arm (31% failure). In addition, a total of 15 children shortened the predefined time window for raw egg tolerance development. In the long arm, 11 children obtained raw egg tolerance within 5–29 months instead of the predefined 30 months while four children in the short arm developed complete tolerance within 5–12 months instead of the predefined 18 months.

### Immunological Parameters

In total, 76 of the 78 children passed the baked egg introduction of whom 55 had an ovomucoid sIgE level ≤ 1.2 kUA/L while 21 children exceeded this cut-off level. Of note, the median age of the latter was 4 years at the time of exposure to baked egg, while the children with ovomucoid sIgE levels ≤ 1.2 kUA/L had a significantly lower median age of 2 years. The six children that had to repeat the baked egg introduction, due to an allergic reaction toward cake (*n* = 2) or hard-boiled egg (*n* = 4), had a median age of 2 years with median IgE levels of 1.47 kUA/L for egg white, 0.72 kUA/L for ovomucoid and 0.11 kUA/L for ovalbumin. The two allergic reactions toward cake consisted of a general increase in facial eczema, and angio-edema combined with diarrhea upon the ingestion of a cupcake that was less heated compared to cake. Probability curves for developing raw egg tolerance within (≤ 18 vs. ≤ 30 months) or beyond (>18 vs. > 30 months) the predefined time window for each arm based on egg white sIgE, ovomucoid sIgE, ovalbumin sIgE, and total IgE were generated (Supplementary Figure S1). ROC curves of these parameters showed areas under the curves of approximately 0.5–0.63 with a *p* > 0.05, indicating that none of the parameters could predict failure in tolerance induction in due time.

## Discussion

We present clinical data supporting the safety and feasibility of our graduated short arm protocol as a home-based treatment option for baked egg tolerant children. This step-wise protocol consecutively introduces egg-containing preparations according to the heating procedure being baked egg offered as cake, hard-boiled egg, omelet, soft-boiled egg, and raw egg. Before designing this graduated protocol, we investigated how different heating temperatures and durations could affect the structure and allergenicity of the egg proteins in these preparations. As expected, SDS-PAGE analyses revealed that heating leads to the disappearance of ovalbumin and ovotransferrin, which is likely attributed to the formation of insoluble aggregates (21, 22). In contrast, ovomucoid was unaffected by thermal processing, fitting with its heat-stable structure. In accordance, ELISA analysis showed that the ovalbumin/total protein ratio increased while the ovomucoid/total protein ratio decreased under lower heating temperature and duration. These observations were in line with results from immunoblots demonstrating their clinical relevance, as the IgE-binding to ovalbumin decreased with extensive heating, while the immunoreactivity to ovomucoid remained unaffected. More interestingly, although heating alone had no effect on the allergenicity of ovomucoid, heating in the presence of wheat proteins led to a decreased IgE reactivity to both ovomucoid and ovalbumin. This is concurrent with earlier observations, attributing this decreased bioavailability of ovomucoid to the formation of insoluble wheat complexes (23–26). In general, these findings confirm our observations in clinical practice on the different preparations of heated egg in the presence or absence of the wheat matrix, being the outcome of the OFC and the clinical evaluation by egg sIgE levels. However, a limitation of our experiments was that we did not re-extract potential heat-aggregated proteins that render insoluble, such as ovalbumin, from the pellet to study their possible allergenic role. Surprisingly, the ovalbumin content of hard-boiled egg was lower compared to the extensively heated oven baked egg. This is contrary to SDS-PAGE results of hard-boiled egg white in previous studies (20, 27, 28). However, as opposed to our SDS-PAGE results, which were further confirmed by ELISA, the bands of ovalbumin and ovomucoid overlapped in previously published work, which could have made it difficult to identify the allergens separately (20, 27, 28).

Based on these findings, we designed our step-wise egg-introducing protocol. When comparing our graduated protocol with existing egg ladders (Supplementary Figure S2), the main difference might be the consecutive step-by-step introduction of hard-boiled egg, omelet, and soft-boiled egg, compared to the one-step introduction defined as “lightly, whole or well-cooked egg” in some of the existing egg ladders (9–11, 29). Hereby, our step-wise introduction was based on the difference in thermal processing and heat-induced modifications of egg allergens in those preparations, as observed by SDS-PAGE and ELISA. Second, we introduce pancakes and waffles along with omelet, while others introduce these preparations earlier based on the “wheat matrix effect” and lower amount of egg per consumption (9–11, 29, 30). Although we also observed this “wheat matrix effect,” we chose to introduce hard-boiled egg in step two due to its low ovalbumin level and longer heating time and decided that solely relying on heating duration (which was ~3' for pancakes, waffles, and omelet) simplified the instructions for parents. Finally, we pre-defined the time period between each step in order to define a clinically efficient time window for egg introduction at home.

Our gradual protocol has proven to be a safe and feasible instrument to guide physicians and parents through the at-home reintroduction of egg. In a baked egg tolerant cohort, the median time for complete raw egg resolution was 24 months in the short arm vs. 30 months in the long arm. Hereby, we consider egg tolerance as a lack of clinical reactivity toward raw egg, while prior studies defined this as tolerance toward lightly cooked egg/nearly raw egg (9–11). Considering that the time to outgrow egg allergy is stated to be 78 months, our study supports the hypothesis that gradually introducing less heated egg products accelerates the resolution of egg allergy (8–10). Several parents also stated that reintroducing egg by our guided protocol increased the quality of life of their child as well as their own. Additionally, we provide new insights into the step-wise tolerance development of hard-boiled egg, omelet, and soft-boiled egg using a guided protocol which was 7 vs. 9 months, 12 vs. 17 months, and 17 vs. 23 months in the short- and long arm, respectively.

One might consider our at-home reintroduction protocol, using heat-induced modifications of egg-containing products, a protocol of “gradual OIT.” The main concern of OIT remains the increased risk of adverse reactions, which have shown to occur in up to 75% of children undergoing classical egg OIT (31). However, we observed that only 11 children (14%) experienced symptoms resulting in the postponement of a next step including facial edema, urticaria, itch, cough, abdominal pain, diarrhea, and/or vomiting, with most of the reactions occurring during the hard-boiled egg introduction. Of these children with adverse reactions, only one child withdrew from the study due to a grade III reaction toward raw egg (final step) at home while the majority of children were able to finalize the protocol. This was also the case for children who experienced eczema flare-ups of which the causal relationship with egg intake remains speculative. Indeed, eczema flare-ups could have been triggered by other exposures, such as a tree- and/or grass pollen (during pollen season) in children experiencing “the Atopic March” (32). Additionally, there were no reports of grade IV reactions toward egg nor OIT–associated clinical signs of EoE (33). Finally, the short arm appeared to be non-inferior to the long arm in relation to adverse events, which further supports the feasibility and safety of the short arm protocol as the most favorable treatment option for baked egg tolerant children. Clinicians could thereby use the 6-month interval between the first steps and 3-month interval between the last steps to guide children through the at-home reintroduction.

One of the limitations of our study is the lack of a control group of egg allergic children strictly avoiding egg-containing products, in order to define whether the protocol really reduces the time to complete tolerance. However, after careful consideration, we decided that including a control group would be unethical, as several studies have already proven that strictly avoiding allergens might be a risk factor for delayed tolerance induction (34). Several reports also documented that it takes a much longer period of 78 months to naturally develop complete egg tolerance (8, 9). Furthermore, early food allergen reintroduction has been shown to improve the quality of life of children and parents, while strict avoidance is rather associated with social limitations, anxiety, and high socio-economic costs (35, 36). A second limitation is that the “permanent state” of this step-wise complete egg tolerance development has not been proven. It is therefore possible that these children's state of unresponsiveness can be temporary (requiring regular exposure) or persistent (independent of regular allergen exposure) (37). However, after completing the study most of the children were further monitored in one of the allergy clinics due to concomitant food allergies and despite no guided protocol for regular intake, these children remained raw egg tolerant. We hereby noticed that without any egg limitation, most children had daily contact with egg-containing products. Thirdly, we focused on a subgroup of baked egg tolerant children with a low baseline median egg white sIgE level of 1.02 kUA/L (between 0.10 and 14 kUA/L) who are known to have a high likelihood of a transient phenotype, while excluding children with persistent baked egg reactivity (38). It would be of utmost importance to unravel the underlying (cellular) immunological changes in this baked egg tolerant group while they become complete egg tolerant, and apply this knowledge to baked egg reactive children. Today, we have limited insight into the underlying immunological profile of these children. To that aim, we are studying the immune phenotypical cellular changes in a new cohort of baked egg tolerant children undergoing the short gradual egg-introducing protocol (NCT04677790).

Finally, we sought to identify immunological predictors of step-wise egg-tolerance development. We found that none of the baseline IgE levels of egg white, ovomucoid, or ovalbumin achieved substantial predictive values (PV) for raw egg tolerance within the predefined time-period. Given that peripheral blood was only collected in case of a clinical indication during follow-up visits, there was not enough data to analyze the changes in the egg sIgE levels throughout the study. Of note, 93% of the children with ovomucoid sIgE levels ≤ 1.2 kUA/L successfully introduced baked egg into their diet, thereby surpassing the previously reported 75% PV (16). Though, also 90% of the children with higher ovomucoid sIgE levels passed their baked egg challenge. The major factor that differed between these two groups was their median age, being 4 years in the latter vs. 2 years, respectively. Moreover, as noted in similar studies, there were some children with undetectable IgE levels for ovomucoid who still reacted during the introduction of baked egg or hard-boiled egg (14–16, 39). Indeed, Lemon-Mulé et al. reported that undetectable ovomucoid sIgE's still have a 10% positive PV for heated egg reactivity (15). Ultimately, these findings are in line with previous studies demonstrating large variations in the prognostic value of egg sIgE levels, accompanied by discrepancies between these IgE levels and SPTs (14, 40–42). The latter of which was not evaluated in our study.

We can conclude that the allergenicity of ovalbumin and ovomucoid is modified by the heating temperature, duration as well as the presence of wheat. Based on these findings, we have designed a gradual egg-introducing short arm protocol allowing clinicians to safely guide baked egg tolerant children toward complete egg tolerance at home. While this protocol might help the majority of baked egg tolerant children, the options in baked egg reactive children remain limited.

## Data Availability Statement

The raw data supporting the conclusions of this article will be made available by the authors, upon request.

## Ethics Statement

The studies involving human participants were reviewed and approved by the Ethics Committee Research of UZ/KU Leuven. Written informed consent to participate in this study was provided by the participants' legal guardian/next of kin.

## Author Contributions

DB, JL, MR, and KC conceptualized and designed the study. DB, JL, MR, SV, KC, KS, LN, and ChaM recruited patients and performed clinical follow-up in function of study visits. ChaM and MD developed the oral food challenge protocols, prepared the egg-containing foods for these oral food challenges, and provided dietary counseling to the parents and children. LDV coordinated the study visits, collected the data and conducted the experiments together with ED and LC. LDV, DB, RS, and ChrM contributed to the design of the experiments, the statistical analysis of the data, and interpretation. LDV drafted the article, which was critically revised and edited by DB. All authors revised the manuscript and approved the final version.

## Funding

DB and RS are recipients of a senior research fellowship from the Fund for Scientific Research Flanders (FWO).

## Conflict of Interest

The authors declare that the research was conducted in the absence of any commercial or financial relationships that could be construed as a potential conflict of interest.

## Publisher's Note

All claims expressed in this article are solely those of the authors and do not necessarily represent those of their affiliated organizations, or those of the publisher, the editors and the reviewers. Any product that may be evaluated in this article, or claim that may be made by its manufacturer, is not guaranteed or endorsed by the publisher.
