# Supplementary material for: Guided Gradual Egg-Tolerance Induction in Hen's Egg Allergic Children Tolerating Baked Egg: A Prospective Randomized Trial
Source: Front Allergy. 2022 May 11;3:886094. doi: 10.3389/falgy.2022.886094 (PMC9234941; doi:10.3389/falgy.2022.886094)
Supplement: Supplementary file 1 [file Data_Sheet_1.docx]

Supplementary Material

# Supplementary figures

**
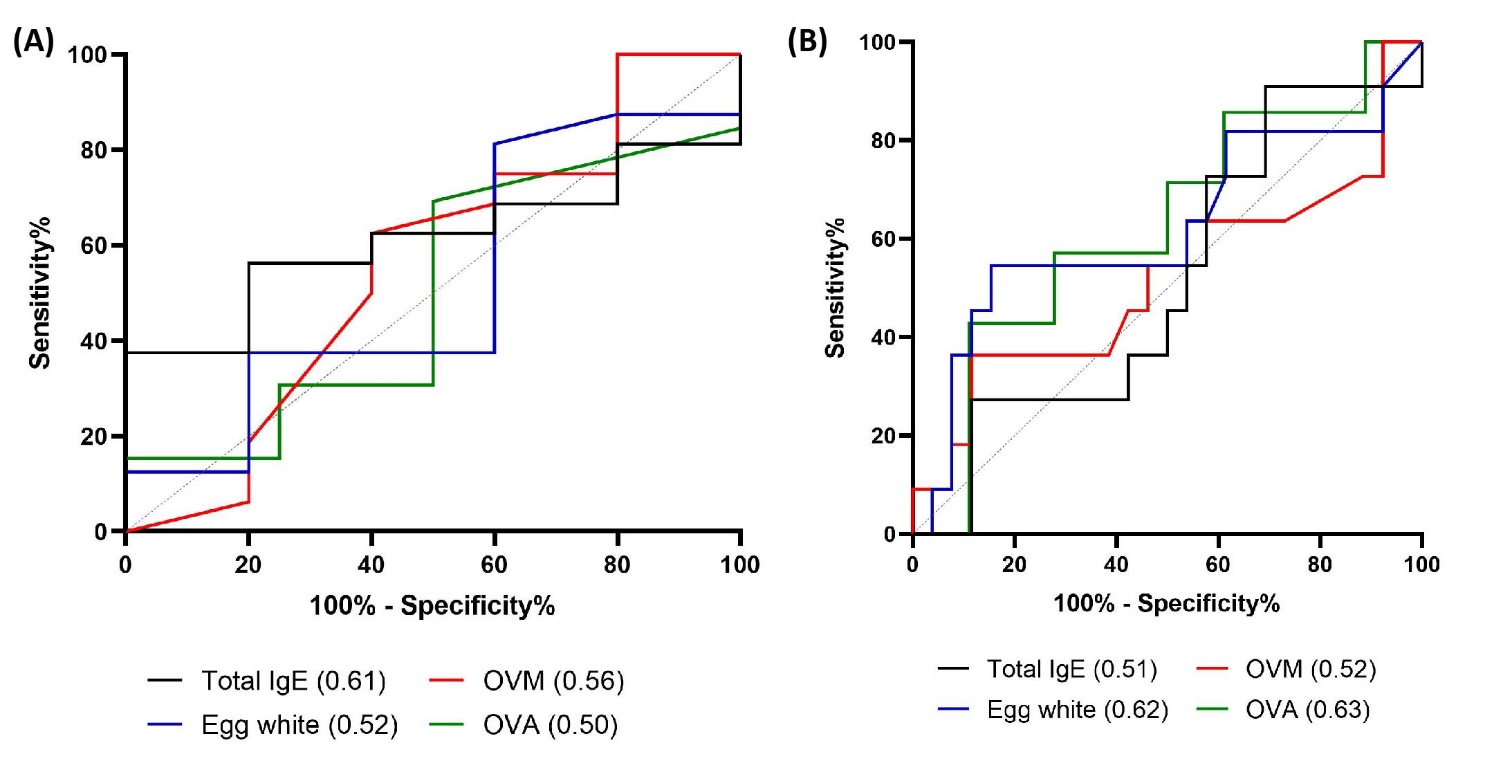
**

**Figure S1:** ROC curves for developing raw egg tolerance based on baseline egg white sIgE, ovomucoid sIgE, ovalbumin sIgE and total IgE **(A)** within (≤ 18 m vs ≤ 30 m) or **(B)** beyond (>18m vs > 30 m) the predefined time window for each arm. OVA: ovalbumin, OVM: ovomucoid.

**
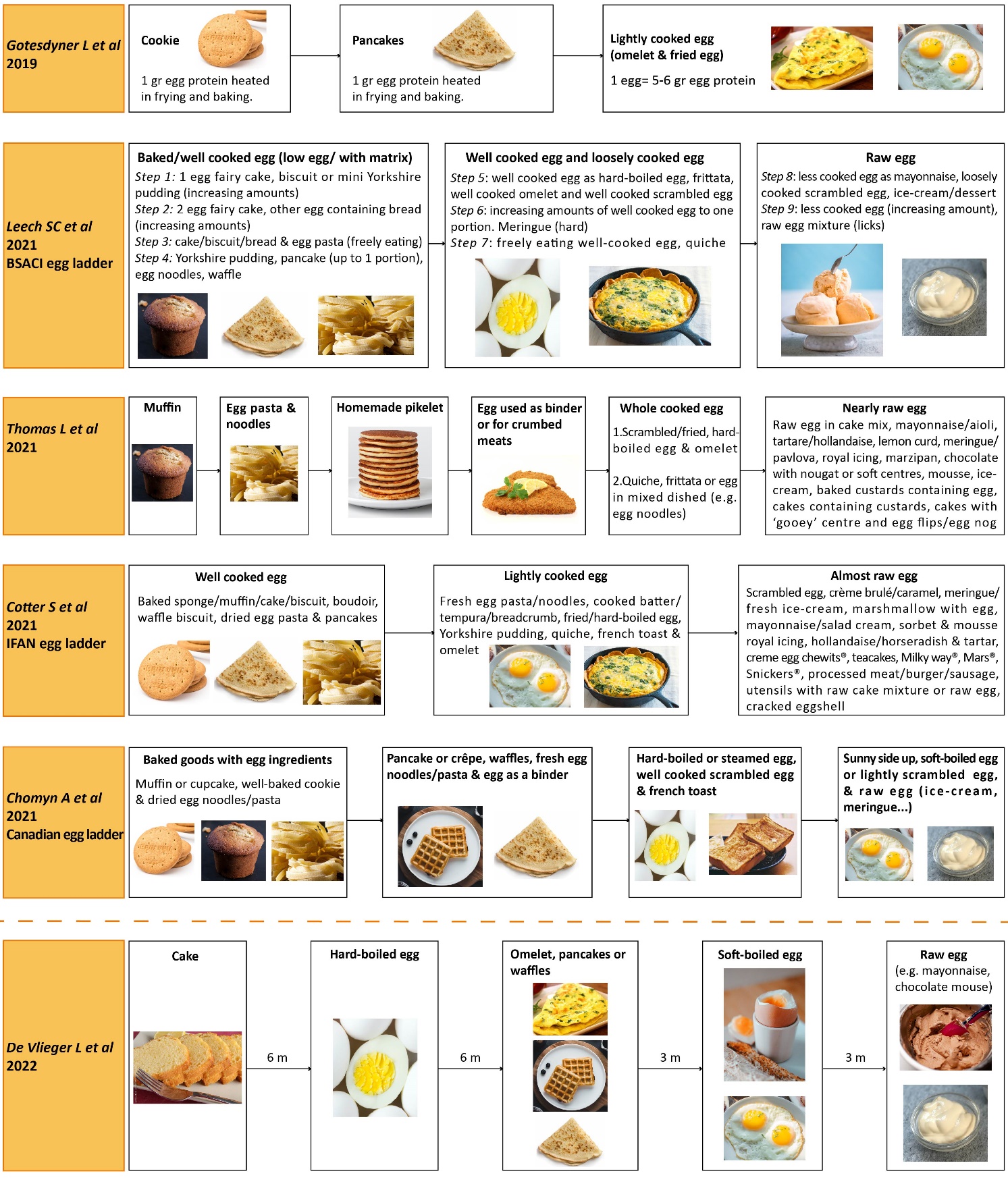
**

**Figure S2:** Brief overview of the existing egg ladders and our short arm graduated protocol. Note: adapted from ref 9 (Fig 1), 10 (Appendix 1), 11 (Appendix 1), 29 (Fig 1) and 30 (Fig 1). Please consult the original articles for more information on the use of these egg ladders.
